# Expression and Function of mARC: Roles in Lipogenesis and Metabolic Activation of Ximelagatran

**DOI:** 10.1371/journal.pone.0138487

**Published:** 2015-09-17

**Authors:** Etienne P. A. Neve, Harald Köfeler, Delilah F. G. Hendriks, Åsa Nordling, Vladimir Gogvadze, Souren Mkrtchian, Erik Näslund, Magnus Ingelman-Sundberg

**Affiliations:** 1 Section of Pharmacogenetics, Department of Physiology and Pharmacology, Karolinska Institutet, Stockholm, Sweden; 2 Medical University Graz, Center for Medical Research (ZMF), Graz, Austria; 3 Institute of Environmental Medicine, Section of Toxicology, Stockholm, Sweden; 4 Department of Clinical Sciences, Danderyd Hospital, Karolinska Institutet, Stockholm, Sweden; East Tennessee State University, UNITED STATES

## Abstract

Recently two novel enzymes were identified in the outer mitochondrial membrane, mARC1 and mARC2. These molybdenum containing enzymes can reduce a variety of *N*-hydroxylated compounds, such as N-hydroxy-guanidines and sulfohydroxamic acids, as well as convert nitrite into nitric oxide (NO). However, their endogenous functions remain unknown. Here we demonstrate a specific developmental pattern of expression of these enzymes. mARC1, but not mARC2, was found to be expressed in fetal human liver, whereas both, in particular mARC2, are abundant in adult liver and also expressed in omental and subcutaneous fat. Caloric diet restriction of obese patients caused a decreased expression of mARC2 in liver, similar to that seen in the livers of starved rats. Knock down of mARC2 expression by siRNA in murine adipocytes had statistically significant effect on the level of diglycerides and on the fatty acid composition of some triglycerides, concomitantly a clear trend toward the reduced formation of most of triglyceride and phospholipid species was observed. The involvement of mARC2 in the metabolism of the hepatotoxic drug ximelagatran was evaluated in hepatocytes and adipocytes. Ximelagatran was shown to cause oxidative stress and knock down of mARC2 in adipocytes prevented ximelagatran induced inhibition of mitochondrial respiration. In conclusion, our data indicate that mARC1 and mARC2 have different developmental expression profiles, and that mARC2 is involved in lipogenesis, is regulated by nutritional status and responsible for activation of ximelagatran into a mitotoxic metabolite(s).

## Introduction

Recently a novel mitochondrial amidoxime reductase enzyme system has been identified in mammals and subsequently the individual components have been characterized. The enzymes were identified based on the assay for amidoxime reductase activity, which was detected in liver, kidney and adipose tissues where the highest specific activity was found to be associated with the outer mitochondrial membrane fractions [1–3]. This enzyme complex was shown to be composed of the electron transport proteins, such as mitochondrial cytochrome b5 type B (CYB5B) and NADH cytochrome b5 reductase (CYB5R) and a third component called the mitochondrial reducing component 1 and 2 (mARC1 and mARC2) [1, 2]. The mARC1 and mARC2 enzymes, previously also referred to as molybdenum cofactor sulfurase C-terminal containing 1 and 2 (MOSC1 and MOSC2) respectively, are two homologous protein members of the MOSC family of proteins. This protein family is responsible for the sulfuration of the molybdenum cofactor present in xanthine dehydrogenase and aldehyde oxidase. However, both mARC1 and mARC2 lack the NifS domain that is responsible for the cysteine sulfurase activity and are therefore unlikely to possess such activity. The mARC1 and mARC2 proteins are encoded by the mARC1 and mARC2 genes and are present in all of the mammalian genomes studied. Human mARC1 and mARC2 are localized in the close proximity on chromosome 1 (location 1q41) only 58 kb apart of each other and at present not much is known about their gene regulation and tissue expression.

The mARC enzyme system is able to reduce a variety of N-hydroxylated compounds such as amidoxime containing prodrugs, N-hydroxy- guanidines and sulfohydroxamic acids. The enzyme system was recently described to be involved in the detoxification of N-hydroxylated derivatives of purines and pyrimidines [4]. The mARC dependent metabolism of N^4^-hydroxy-L- arginine, a known precursor of NO biosynthesis may suggest a role in the regulation of nitric oxide (NO) biosynthesis. Furthermore, both mARC1 and mARC2 have been shown to reduce nitrite to NO [5]. However, the true endogenous substrates and therefore the physiological role of this mitochondrial enzyme system is not clear. We have previously suggested that mARC2 might be involved in lipid synthesis or catabolism, since mRNA and protein levels of mARC2 (but not mARC1) were increased during adipogenesis in a murine adipocyte cell model [3]. Moreover, down- regulation of mARC2 in mature adipocytes decreased the intracellular triglyceride levels, suggesting that mARC2 could be involved in lipogenesis. In addition, animal studies demonstrated that the activity mARC enzymes can be affected by fasting and high-fat-diet [6].

Clinical trials of the thrombin inhibitor ximelagatran (Exanta®), revealed that it is hepatotoxic in 7% of Caucasian patients as indicated by the increased levels of circulating AST [7]. Subsequent to the intake, the prodrug is converted in two steps to the active form melagatran [8]. Because of the amidoxime moiety in the compound it has been proposed that ximelagatran is a substrate for mARC (cf. [3, 9]). It can therefore be hypothesized that the hepatotoxicity of ximelagatran is connected with the mARC dependent conversion of this drug into melagatran.

In the present study we have mapped the developmental expression profile of these enzymes. In addition, the expression of mARC1 and mARC2 in human subcutaneous and omental fat from obese individuals was found to be down regulated upon starvation. The role of mARC2 in lipid synthesis and metabolic activation of ximelagatran was evaluated in differentiated adipocytes subsequent to siRNA-mediated knock down. The results indicate that mARC2 is regulated by caloric restriction in man, can contribute to lipid metabolism and is responsible for activation of ximelagatran into mitotoxic endproducts.

## Materials and Methods

### Human subjects

A standard laparoscopic Roux-en-Y gastric bypass (RYGB) with a 1 m Roux-limb was performed. The patients were weight stable and either not subjected to a preoperative weight loss period or underwent a two week 1000 kcal/day preoperative very low calorie diet. Subcutaneous and omental abdominal adipose and liver biopsies (50–100 mg) were obtained at the beginning of RYGB surgery after the induction of general anesthesia. Only non- glucose-containing intravenous solutions were administered before the biopsy was taken during RYGB surgery after an overnight fast. The study was approved by the Regional Ethics Committee of Stockholm (2010/1046). The patient characteristics are presented in Table 1.

**Table 1:** Clinical characteristics of the obese patients.

Patient	BMI	Gender	Fasted	Weight	YOB
1	38	F	Y	1–3 kg	1966
2	42	F	Y	1–3 kg	1950
3	37	M	Y	1–3 kg	1984
4	34	F	Y	5 kg	1976
5	34	F	Y	7 kg	1977
6	34	M	Y	3 kg	1978
7	39	F	Y	7 kg	1975
8	36	F	N	-	1980
9	37	M	N	-	1990
10	41.5	F	N	-	1976
11	35	F	N	-	1957
12	39	F	N	-	1976
13	35	F	N	-	1972
14	41	F	N	-	1976
15	51.5	F	Y	+1.5 kg	1986
16	37	F	Y	-	1953

### Human adult and fetal liver tissue

Liver samples from fourteen 8–12 weeks old fetuses were acquired from Karolinska University Hospital biobank (Huddinge, Sweden). Eighty- eight liver samples were from adult organ donors who met accidental death or from patients undergoing resection due to malignant extra hepatic tumors. Of them, 70 were acquired from Karolinska University Hospital, and 18 were commercially purchased from XenoTech (Lenexa, KS) and the International Institute for the Advancement of Medicine (IIAM, Edison, NJ).

This work was carried out in compliance with the methods within the Helsinki declaration. The use of fetal and adult liver tissues for the purposes of this study was approved by the Regional Ethics Committee of Stockholm (2010/541-23/1, 2010/541-31/1, 2010/678-31/3 and 280/00).

RNA from these tissues was isolated using the AllPrep DNA/RNA/Protein Mini Kit (Qiagen). The TargetAmp™-Nano Labeling Kit for Illumina® Expression BeadChip (Epicentre, Chicago, IL) was used to amplify and biotinylate RNA, according to the manufacturer’s instructions. Biotinylated cRNA was hybridized to HumanHT-12 BeadChips (Illumina, v4 arrays, San Diego, CA), according to the standard protocol. The BeadChips were scanned within 24 h using a HiScanSQ scanner. The raw signals were exported using GenomeStudio (Illumina).

### RNA isolation, cDNA synthesis and real time PCR analysis

RNA was isolated from 100–150 mg subcutaneous or omental human fat tissue and 20 mg of the corresponding liver biopsy using the RNA Easy Midi kit (Qiagen). For the human fetal and adult livers, RNA was isolated as indicated above. cDNA was synthesized from 0.1 μg RNA using SuperScript® III First-Strand Synthesis SuperMix (Invitrogen) with Oligo(dT) primers. cDNA was subjected to quantitative real time PCR (qRT-PCR) analysis to quantify mRNA levels of mARC1 and mARC2 using the TaqMan Gene Expression Assays Hs00224227_m1 and Hs00215486_m1, respectively (Invitrogen). As a housekeeping gene the TATA box binding protein (TBP) was used for normalization. The relative expression levels were defined by the 2^-ΔΔCt^ method as described elsewhere [10].

### Subcellular fractionation of the human subcutaneous and omental fat and liver tissues

Subcutaneous and omental fat tissue and liver biopsies were homogenized using a Bullet Blender (Next Advance Inc., Averill Park, NY). 1.0 g of subcutaneous or omental fat tissue was homogenized in 1.5 ml of the tissue homogenization buffer (100 mM Phosphate pH 7.4, 150 mM NaCl, 250 mM sucrose, 1 mM EDTA, supplemented with protease inhibitors) containing 0.5 g zirconium oxide beads (2.0 mm) in the Bullet Blender (Speed 8, 3 times 1 min) at 4°C. Debris, nuclei and the zirconium beads were settled at 800 x g for 5 min and the supernatant was transferred to the new tubes. The supernatant was centrifuged at 10,000 x g for 10 min and the pellet, mitochondrial (P10) fraction was re-suspended in homogenization buffer, and the supernatant (S10) was collected and protein concentration determined according to Bradford. 50 mg of the matching liver tissues were homogenized in 0.2 ml tissue homogenization buffer using 50 mg zirconium oxide beads (0.5 mm) and processed as described above.

### Western blot analysis

Equal amounts of protein from the isolated P10 and S10 fractions (for the analysis of fat tissues and matching livers) or from the protein fractions from the fetal and adult livers isolated using the AllPrep DNA/RNA/Protein Mini Kit (Qiagen) were subjected to SDS polyacrylamide gel electrophoresis and the separated proteins were transferred to nitrocellulose membranes. The membranes were routinely Ponceau stained to verify that equal amounts of lysates were loaded. mARC1 polyclonal antibodies were purchased from Sigma-Aldrich (cat. # SAB4301192) and mARC2 polyclonal antibodies from Atlas Antibodies, Stockholm, Sweden (cat. # HPA015085). In addition we also used polyclonal anti-ERp29 antibody [11] and monoclonal mitochondrial heat shock 70 (mHSP70) antibody (Affinity Bioreagents, Thermo Scientific). The secondary antibodies were conjugated with HRP (Dako Denmark A/S, Glostrup, Denmark). The blots were developed using SuperSignal West Pico Chemiluminescent Substrate (Thermo Scientific) and visualized using a Fuji Las-1000+ luminescent image analyzer (FujiFilm).

### Differentiation of 3T3-L1 cells and knock down of mARC2 expression

Culturing of murine 3T3-L1 pre-adipocytes, differentiation into adipocytes and mARC2 siRNA transfections were described in detail elsewhere [3].

### Animal study

Male Sprague-Dawley rats (150–200 g, Anticimex, Stockholm, Sweden) were used in this study. The control animals had free access to food, while in the starvation group three animals were deprived of food and starved overnight; animals in both groups had free access to drinking water. Animals were killed by asphyxiation using CO_2_, livers were excised and the mitochondrial fraction was isolated as described previously [1]. The animal studies were performed in 2005 and approved by the local animal ethical committee in Stockholm, Sweden.

### Amidoxime reduction assay

The amidoxime reductase activity assay was performed as described previously [3].

### Analysis of cell lipidome

Cell pellets (3–4 x 10^6^ cells) were suspended in 1.5 ml of methanol and lipids were extracted with the MTBE method according to Matyash et al. [12]. Lipid extracts were re-suspended in 200 μl CHCl3/MeOH 1:1 containing 200 pmol of PC 12:0/12:0 as non- endogenous internal reference compound. Mass spectrometric data acquisition was performed as described in more detail previously [13]. Briefly, 1 μl was injected into a Thermo Hypersil GOLD C18, 100 x 1 mm, 1.9 μm HPLC column used with an Accela HPLC system (Thermo Scientific). Solvent A was water with 1% ammonia acetate and 0.1% formic acid, and solvent B was acetonitrile/2-propanol 5:2 with 1% ammonia acetate and 0.1% formic acid. The gradient ran from 35% to 70% B in 4 min, then to 100% B for another 16 min where it was held for 10 min. The flow rate was 250 μl/min. Data acquisition was done by Orbitrap mass spectrometry (LTQ-Orbitrap, Thermo Scientific) full scan in preview mode at a resolution of 100k and < 3 ppm mass accuracy with external calibration. The spray voltage was set to 5000 V, capillary voltage to 35V and the tube lens was at 120V. Capillary temperature was at 250°C. From the Orbitrap-MS preview scan the 10 most abundant m/z values were picked in data dependent acquisition (DDA) mode fragmented in the linear ion trap analyzer and ejected at nominal mass resolution. Normalized collision energy was set to 35%, the repeat count was 2 and the exclusion duration at 60 s. Data analysis was done by Sieve software (Thermo Scientific) online gated to Lipid MAPS Structure Database (LMSD). Annotation of lipid species is according to the official Lipid MAPS shorthand nomenclature [14].

### Analysis of oxygen consumption

Mitochondrial respiration was monitored with an oxygen electrode (Hansatech Instruments, Norfolk, UK) and analyzed with the OxygraphPlus software (Hansatech Instruments). After harvesting the differentiated 3T3-L1 adipocytes (3 × 10^6^) were re-suspended in 300 μl of culture medium and added to the respiration chamber. After monitoring basal oxygen consumption, 5 μM of the uncoupler, carbonyl cyanide m-chlorophenyl hydrazine (CCCP) was added to dissipate the mitochondrial membrane potential and the maximal activity of the mitochondrial respiratory chain was assessed.

### Primary human hepatocyte experiments

Freshly isolated primary human hepatocytes (PHHs) were seeded in the plates pre-coated with rat-tail collagen I (Corning). PHHs were cultured in William’s medium E supplemented with 100 U/ml penicillin, 100 μg/ml streptomycin, 2 mM L- glutamine, 1.7 μM insulin, 5.5 μg/ml transferrin, 6.7 ng/ml sodium selenite, 100 nM dexamethasone and 10% fetal bovine serum (Gibco). After cell attachment, PHHs were shifted to the same medium but without serum. Cells were kept under sterile conditions at 37°C, 95% air humidity and 5% CO_2_.

### PHH viability

Cell viability was assessed by measuring the cellular ATP content using the CellTiter-Glo Luminescent Cell Viability Assay (Promega). Luminescence was measured using a MicroBeta^2^ LumiJET luminometer (PerkinElmer).

### GSH content

Total glutathione (GSH + GSSG) and oxidized glutathione (GSSG) levels in PHH were assessed using the GSH/GSSG-Glo Assay (Promega). GSH levels were calculated by multiplying the GSSG level by two and subtracting this value from the total glutathione level.

### Thioredoxin 2 redox state analysis

The redox state of the mitochondria-specific thioredoxin 2 (Trx2) in control- and ximelagatran exposed PHHs was assessed using redox Western blotting as described by [15, 16] with some modifications. In brief, cells were harvested, washed with cold PBS and re-suspended in 300 μl lysis buffer (50 mM Tris-HCl, 1 mM EDTA, 8 M urea (pH 8.3)) supplemented with 30 mM iodoacetamide (IAM) to alkylate free sulfhydryl groups. Proteins were precipitated by ice-cold acetone/1 M HCl (98:2 v/v). The precipitate was solubilized in 100 μl lysis buffer containing 3.5 mM dithiothreitol to reduce disulfide bonds, which were subsequently alkylated by the addition of 2 μl 600 mM iodoacetic acid (IAA). Protein concentrations were determined by the bicinchoninic acid assay (Pierce). Equal amounts of protein were separated on a 12% urea-PAGE gel. Reduced Trx2 migrates slower than oxidized Trx2, because the free sulfhydryl groups of reduced Trx2 are alkylated with IAM, whereas the sulfhydryl groups of oxidized Trx2 are alkylated with IAA, which is more negatively charged.

Proteins were blotted to a nitrocellulose membrane (Amersham) and blocked with 5% bovine serum albumin. Trx2 was detected by polyclonal anti- Trx2 antibody (1:1500, Santa Cruz) and HRP conjugated secondary antibodies (1:2000, Dako). SuperSignal West Pico Chemiluminescent Substrate (Pierce) was used for detection and chemiluminescence was assessed with ChemiDoc XRS+ System (Bio-Rad). Image Gauge software was used for densitometric analysis of protein bands.

### Data analysis

Statistical analysis was carried out using the GraphPad Prism v5 software. Data were analyzed using unpaired t test or one- way analysis of variance with use of Dunnett’s multiple comparison test or Bonferroni’s multiple comparison test.

## Results

### Expression of mARC1 and mARC2 in human fetal and adult liver

**Fig 1:**
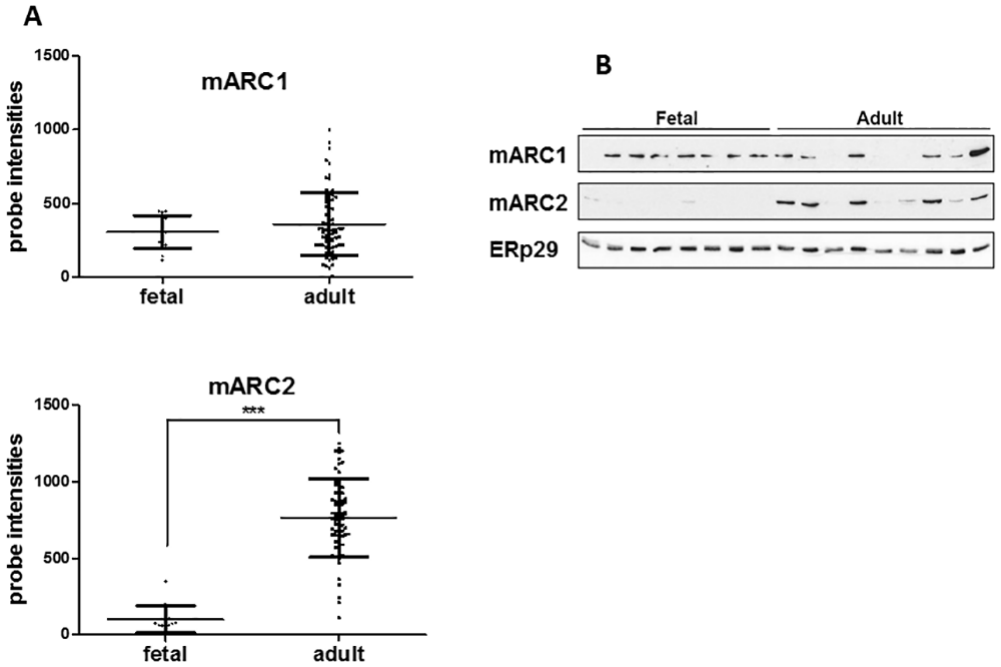
Expression of mARC1 and mARC2 in human adult and fetal liver. A. mARC1 and mARC2 gene expression in human fetal (n = 14) and adult (n = 88) liver samples was determined by microarray analysis and presented as background corrected probe intensities. ***p<0.001. B. mARC1 and mARC2 protein expression in 8 fetal and 9 adult livers. Equal amounts of protein were analyzed for the expression of mARC1 (upper panel), mARC2 (middle panel). Specific bands for both proteins were identified at the level of approximately 35 kDa, an apparent molecular mass of mARC1 and 2. ERp29 was used as a loading control (lower panel).

In order to examine the developmental pattern of the expression of mARC1 and mARC2, the mRNA levels of both genes were analyzed in 88 adult and 14 fetal human livers (S1 Fig, Fig 1A). The mARC1 mRNA expression levels appear not to be significantly different between the fetal and the adult tissues, although the variability is larger in the adult livers compared to the fetal ones. In contrast, mARC2 is only expressed in the adult livers, only very low levels are observed in the fetal tissues, suggesting that the expression of mARC2 is developmentally regulated while the expression of mARC1 is not.

In order to confirm the expression of mARC2 and mARC1 on the protein level, the several adult and fetal liver samples used in the mRNA analysis were analyzed by Western blot (Fig 1B). In line with the mRNA expression data, mARC1 is detected in both the adult and fetal livers, although there seems to be a considerable variation in both the fetal and adult tissues. mARC2 protein is exclusively expressed in the adult livers and no significant protein levels could be detected in the fetal samples (Fig 1B). As it is observed for mARC1, the mARC2 expression profile also displays significant interindividual variability.

### Expression of mARC1 and mARC2 in omental and subcutaneous fat tissues and livers from obese patients

**Fig 2:**
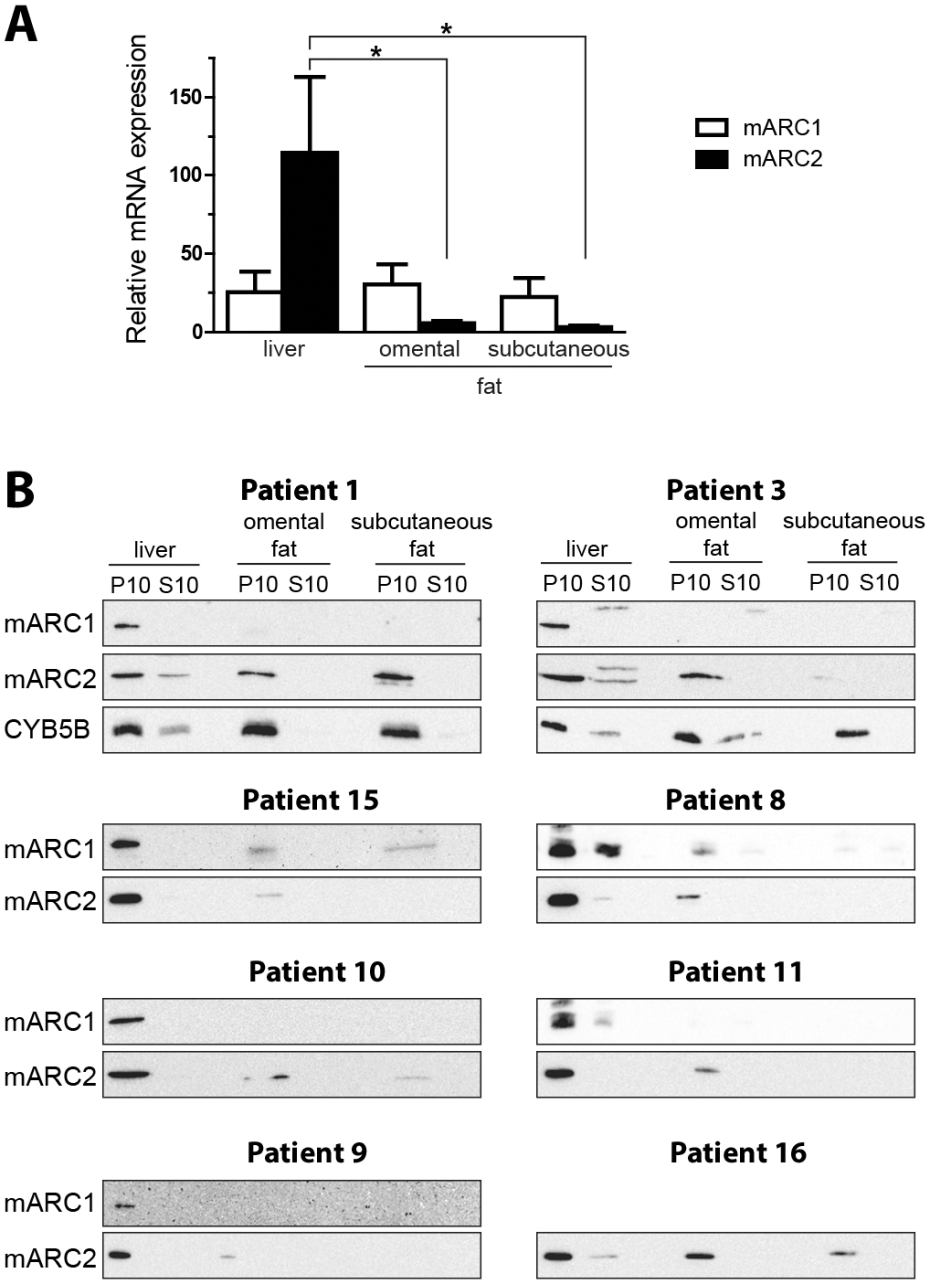
Expression of mARC1 and mARC2 in human subcutaneous and omental fat tissues from obese patients. A. mARC1 and mARC2 gene expression in human subcutaneous and omental fat tissues (n = 6) and matched liver samples (n = 8) in obese patients as determined by qRT-PCR analysis. The Ct values were normalized to the housekeeping gene TBP. Data are expressed as mean ± S.D., *p<0.05. B. Expression of mARC1 and mARC2 proteins in the subcellular and omental fat tissue and matched liver samples in 8 obese patients (patient data are presented in the Table 1). The mitochondrial (P10) and post- mitochondrial (S10) fractions were isolated from the different tissues and analyzed by western blot for the presence of mARC1 and mARC2. Mitochondrial cytochrome b5 type B (CYB5B) is included as a mitochondrial marker protein.

In the previous study we demonstrated that high levels of mARC2 associated amidoxime reductase activity could be detected in rat adipose tissue [1]. Moreover, mARC2 protein expression and the corresponding amidoxime reductase activity were detected in a murine adipocyte cell model [3]. This prompted us to investigate the mARC2 as well as mARC1 levels in the human fat tissues. For this purpose, both omental and subcutaneous fat tissues, together with the liver samples, were collected from obese patients undergoing gastric bypass surgery. Both fat tissues and the matched liver biopsies were analyzed for mARC1 and mARC2 mRNA and protein expression (Fig 2). The mean mARC1 mRNA levels appear not to be significantly different in all of the three analyzed tissues (Fig 2A). In contrast, the mARC2 mRNA levels are significantly higher in the liver as compared to the both fat tissues. Thus, the expression of mARC2 mRNA in the omental and subcutaneous fat is 20-30-fold lower than that of the liver. The omental and subcutaneous fat and the liver samples were fractionated to obtain mitochondrial pellet (P10) and post-mitochondrial supernatant containing endoplasmic reticulum and cytosol and both subcellular fractions were analyzed for mARC1 and mARC2 protein expression (Fig 2B). Both proteins are associated with the mitochondrial fraction (P10) with little or no protein present in the post-mitochondrial supernatant (S10), demonstrating that also in fat tissues these proteins are localized in mitochondria as it was shown earlier for liver [3, 17]. In general, the levels of both proteins and especially the mARC1 are lower in the fat tissues as compared to the matching livers, although a considerable variation can be observed. The mARC2 expression in the omental and subcutaneous fat is comparable to the liver levels in patient 1, whereas in patients 15 and 9 the mARC2 it is barely detectable. Other individuals show intermediate mARC2 expression, but levels in the omental fat are in general higher than those in the subcutaneous fat. The mARC1 levels are considerably lower in both fat tissues, low mARC1 levels can be observed in two patients (15 and 8), while in the others no significant protein expression could be detected, indicating that mARC1 is poorly expressed in omental and especially in subcutaneous fat tissue.

### mARC2 levels are dependent on the nutritional status

**Fig 3:**
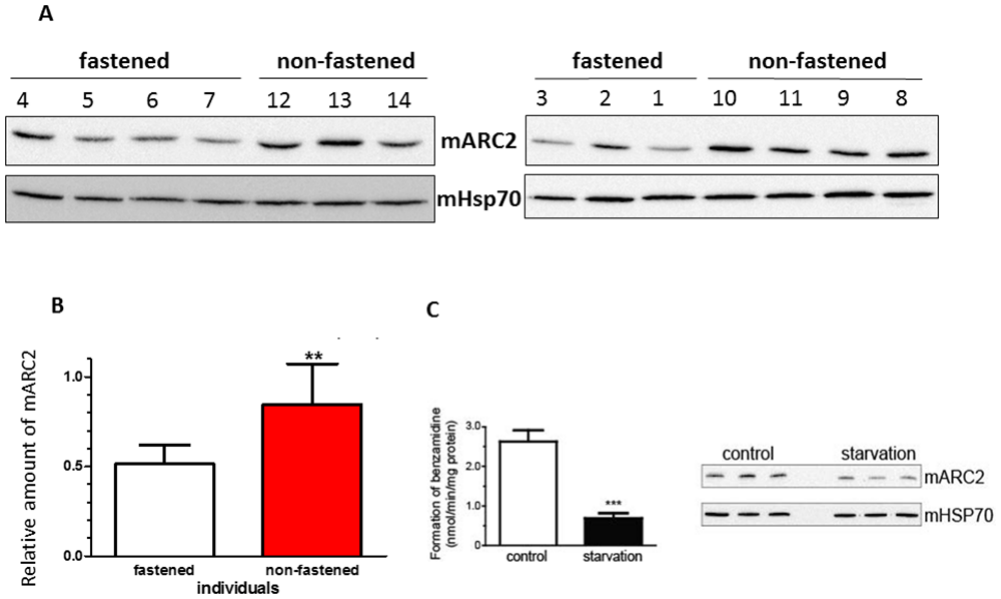
Nutritional status in both human and rats affects the mARC2 levels. A. mARC2 protein levels in obese patients that were put on a caloric restriction diet (fasted) prior to surgery (n = 7) and control (non-fasted) individuals (n = 7) (patient data are presented in the Table 1). Equal amounts of protein from the hepatic mitochondrial fractions were analyzed by western blot for mARC2 and loading control, mitochondrial heat shock protein 70 (mHSP70) (lower panels). B. Both bands were quantified by densitometric analysis and mARC2 levels were normalized by mHSP70. The results represent the mean ±S.D. (n = 7 in each group), **p<0.01. C. mARC2 protein levels and the associated amidoxime reductase activity are decreased in the starvation treated rats. The mitochondrial fractions from the livers from control (n = 3) and starvation treated (n = 3) animals were analyzed for amidoxime reductase activity using benzamidoxime as a substrate (left panel). The results are presented as the mean ± S.D. values. ***, p<0.001. The mitochondrial fractions from these animals were also analyzed by western blot for the presence of mARC2 (right panel). mHSP70, mitochondrial chaperone was used as a loading control.

The relatively high expression of mARC2 in fat tissues and a positive correlation between the mARC2 levels and adipocyte differentiation [3] suggests possible involvement of this enzyme in lipogenesis. Therefore we decided to determine the mARC2 protein levels in the liver samples from fasted and non-fasted obese patients. The fasted patients were put on a caloric restriction diet prior to surgery and were all confirmed to have lost weight between 1 and 7 kg (Table 1). As it is evident from the western blot images, the mARC2 levels in the fasted patients are lower than the protein levels in non-fasted patients who were allowed to continue their normal diet (Fig 3A). This is confirmed by the densitometric analysis of the protein bands showing about 40% lower expression of mARC2 in the liver biopsies from fasted patients (Fig 3B).

As mARC2 levels in the liver are affected by the caloric restriction diet in humans, we decided to examine both the mARC2 protein levels and the corresponding amidoxime reductase activity in livers from starvation treated and control rats (Fig 3C). Western blot analysis of the mitochondrial fractions isolated from the rat livers as well as the analysis of mARC2 dependent reduction of benzamidoxime to benzamidine reveals that both the protein expression (Fig 3C, right panel) and the corresponding catalytic activity (Fig 3C, left panel) are decreased in the starved animals. These results confirm that the mARC2 levels and the corresponding amidoxime reductase activity are dependent on the nutritional status.

### Lipid profiles in mARC2 down-regulated adipocytes

We further explored possible role of mARC2 in the lipid biosynthesis by using an RNAi approach with the extensive analyses of different classes of lipids using mass spectrometry (MS). Within the TG lipid class the most abundant species contain 50 fatty acyl carbons with one two or three double bonds (S2 Fig). This indicates a combination of palmitic, palmitoleic and oleic acid as the major set of fatty acids. mARC2 siRNA treatment caused a clear overall trend towards a decreased amounts of lipids, with two exceptions with the statistically significantly higher TG amounts in mARC2 knock down cells. These are the species TG 38:1 (fold change 3.6) and TG 38:2 (2.8). In contrast, siRNA treatment significantly (0.5) decreased the level of TG 52:0 (Table 2). Analysis of the constituent fatty acids by MS/MS spectra interpretation shows very unusual and asymmetric fatty acids compositions for both 38 carbon species. They contain two long chain fatty acids (C16 or C18) combined with short chain fatty acids butyric, caproic, valeric and even acetic acid.

**Table 2:** Statistical analysis of the mARC2 downregulation effect on specific lipids in the differentiated adipocytes (TG, triglycerides; DG, diglycerides).

Species	Ratio siRNA/control	StdDev	p
TG 38:1	3,60	2,76	0,00017
TG 38:2	2,76	1,99	0,00064
TG 52:0	0,50	0,19	0,00078
DG 42:10	0,76	0,2	0,021
DG 42:11	0,75	0,24	0,031

Phospholipids showed the same trend as TG species (S3 Fig). The suppression of the mARC2 expression results in the uniform trend, though not statistically significant, toward the decrease of the levels of all of the phospholipid classes studied.

The DG lipid class shows only DG 42:10 and DG 42:11 and both of them are significantly down regulated in mARC2 knock down cells (Table 2). They presumably contain very long chain polyunsaturated fatty acids like arachidonic, eicosapentaenoic or docosahexaenoic acid. The available data on free fatty acids show that they remain unchanged between mARC2 knock down and control adipocytes (results not shown).

### Analysis of mitochondrial respiration and cell redox state

**Fig 4:**
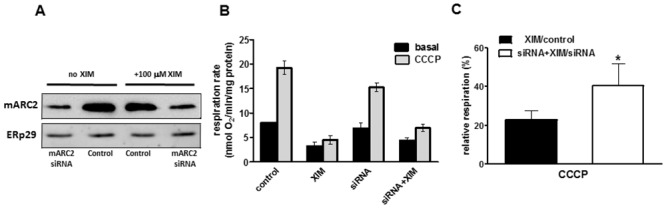
Basal and uncoupled mitochondrial respiration of adipocytes treated with mARC2 siRNA and ximelagatran. A. mARC2 expression in the control and siRNA treated differentiated adipocytes in the presence and absence of 100 μM ximelagatran. B. Basal and CCCP-stimulated mitochondrial respiration in the control or mARC2 knocked down adipocytes in the presence or absence of ximelagatran. C. Remaining respiration after treatment with ximelagatran was expressed as a percentage of corresponding untreated samples. (n = 3). *, p<0.05.

Although the endogenous function of the mARC enzymatic system remains unknown, its mitochondrial localization and N- reductive catalytic activity using NADH as a source of electrons might suggest a possible cross-talk/competition with a mitochondrial respiratory chain. We attempted to address this issue by measuring the oxygen consumption in the differentiated adipocytes with a suppressed expression of mARC2. The siRNA transfected cells demonstrate slightly lower, albeit not supported statistically level of the respiratory activity (Fig 4B). In order to investigate whether the mARC function is connected/interferes with the free electron transport not coupled with the oxidative phosphorylation, the cells were treated with the CCCP, a protonophore causing mitochondrial depolarization and therefore uncoupling respiration from the ATP synthesis. As expected, this significantly increased the oxygen consumption and we also observed a similar trend towards a slight decrease of respiration in the siRNA transfected cells (Fig 4B). As the endogenous function of the mARC enzymatic system in the differentiated adipocytes is not known, we decided to challenge the cells with the putative exogenous substrate of mARC2, ximelagatran. Treatment of adipocytes caused a significant suppression of oxygen consumption, both basal and stimulated by the CCCP (Fig 4B).

Interestingly, the uncoupled respiration (in the presence of CCCP) measured in relation to the untreated samples was significantly higher in the cells with the inhibited expression of mARC2 (Fig 4C). Contrary to the basal oxygen consumption that in addition to the respiratory chain complexes depends on many other factors including oxidative phosphorylation and membrane potential, the uncoupled respiration reflects exclusively the activity of the respiratory chain and hence its alteration, mostly of toxic origin, indicates interference with the various chain complexes. Therefore the recovery of CCCP dependent oxidation in the siRNA treated cells indicates an important role of mARC2 in converting ximelagatran into a putative cytotoxic metabolite(s) that target the mitochondrial respiratory chain.

**Fig 5:**
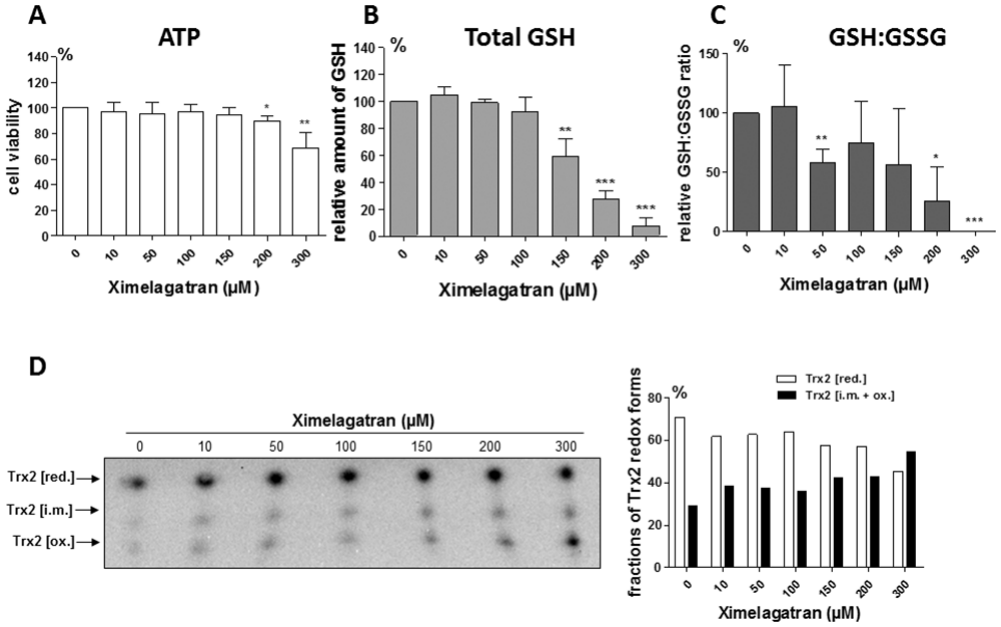
Redox state changes in primary human hepatocytes treated with ximelagatran. A. Effect of ximelagatran treatment on the cell viability (cellular ATP content) and the cellular GSH (B) and GSH:GSSG ratio (C) in PHHs derived from three different donors. D. Analysis of the redox state of mitochondrial-specific Trx2 in control- and ximelagatran-treated PHHs by redox Western blotting. Densitometric analysis of the fractions of different Trx2 redox forms is shown on the right panel. Data are representative for one donor. *, p<0.05; **, p<0.01: ***, p<0.001.

The mitochondrial toxicity of the mARC2 mediated ximelagatran activation was further studied in the primary human hepatocytes (PHHs). After 24 hour ximelagatran exposure, the hepatocyte viability, as assessed by the cellular ATP content, started to decrease at 200 μM concentration (Fig 5A). This was accompanied with a drop in total cellular GSH content (Fig 5B) as well as with reduction of the GSH-GSSG ratio already at the 50 μM concentration indicating conditions similar to oxidative stress (Fig 5C). The mitochondrial redox state was also found altered upon ximelagatran treatment as to the mitochondria-specific thioredoxin 2 (Trx2) oxidation test. Using redox Western blotting fully reduced Trx2 (Trx2 [red.]) can be distinguished from partly oxidized Trx2 (Trx2 [i.m.]) and fully oxidized Trx2 (Trx2 [ox.]), due to differences in the electrophoretic migration. Ximelagatran exposure caused a rapid shift from the fully reduced Trx2 to its partly and fully oxidized forms, which became apparent at 150–200 μM concentrations of the drug (Fig 5D). These results indicate that ximelagatran can alter the mitochondrial and in general cellular thiol redox state and these changes occur before the decrease in cell viability becomes apparent.

## Discussion

In the present investigation we have detailed the developmental pattern of expression of two mARC enzymes and their possible role in lipid metabolism. The developmental regulation in the liver was found quite distinct with mARC1 being expressed in both adult and fetal tissues (more in fetal) while mARC2 is mostly present in the adult liver. Our previous study showed that concomitantly with differentiation of murine 3T3-L1 cells into mature adipocytes, the mARC2 levels were increased, indicating possible involvement of the enzyme in the lipid anabolism. This is consistent with the present data showing relatively high levels of mARC2 expression in the different human adipose tissues, preferentially in the omental fat.

Furthermore, a role in lipogenesis appears to be connected with a regulation by the nutritional status. Indeed, we found a reduction of mARC2 levels in liver in obese patients that were put on a caloric restriction diet. In addition, data from the control and starvation treated rats showed similar results, supporting previous data obtained from mice [6].

Analysis of the cell lipidome in the differentiated adipocytes after siRNA mediated down regulation of mARC2 indicated a significant down regulation of diglycerides and a general, but not significant, trend toward a decrease of most of the triglyceride and phospholipid species. As the cell number upon mARC2 knock down remains constant this might reflect a slightly diminished lipid droplet size or number. Assuming the importance of phospholipids as structural and functional constituents of biomembranes and the lipid droplet coating monolayers, the concerted decrease trend for both lipid classes is not surprising [3]. Nevertheless, one would need additional metabolic flux studies with stable isotope labeled substrates to understand if the diminished lipid amounts is either due to a decreased fatty acid uptake and storage, or due to the non-compensated increased cellular fatty acid export rate.

In spite of the general decrease trend, two of the relatively less abundant triglycerides, TG 38:1 and TG 38:2, both containing one short chain fatty acid, where found significantly upregulated (Table 2). One explanation here would be the accumulation of acetic, butyric and caproic acid due to a lowered mitochondrial short chain fatty acid β–oxidation rate, which is dependent on the short chain specific acyl-CoA dehydrogenase [18], thereby suggesting a specific effect of mARC2 on this enzyme.

Despite the general trend for down regulation for most of the lipid classes upon the mARC2 knock down, the lack of specificity indicates that the putative involvement of mARC2 in the lipid synthesis is indirect and could apparently be accomplished by influencing critical redox systems responsible for lipogenesis. The identity of these factor(s)/system(s) remains to be elucidated.

While the endogenous redox function of mARC enzymes is still unknown, the reduction of a variety of N-hydroxylated exogenous substrates has been firmly established. In particular, the conversion of a prodrug, ximelagatran to the active component melagatran [8] has been suggested to involve a mARC catalyzed reaction [3, 9]. During the development of this drug it was discovered that in some instances it can be hepatotoxic [7, 19]. The mechanisms behind this toxicity are unknown.

Interestingly, it was demonstrated that carriers of the HLA alleles DRB1(*)07 and DQA1(*)02 are at a higher risk for ximelagatran induced cytotoxicity [20], although this association, based originally on a rather limited number of patients was not reproduced. The mechanisms of such idiosyncratic type of drug induced hepatotoxicity are not well investigated. However, it is clear that for many drugs causing HLA and immune dependent toxicity, disruption of a mitochondrial function is quite common event and probably a central point for the drug induced idiosyncratic hepatotoxicity [21]. The finding of the presence of mARC2 specifically in the outer mitochondrial membrane (3) thus lead us to hypothesize that the toxicity of ximelagatran could be mediated by this enzyme and have mitochondria as a primary target. Indeed, we found that siRNA meditated down regulation of mARC2 in differentiated adipocytes significantly recovered the ximelagatran dependent inhibition of mitochondrial respiration (Fig 4). In addition, experiments using primary human hepatocytes showed that ximelagatran induced a decrease in the cellular GSH:GSSG ratio as well as stimulated the oxidation of the mitochondria specific thioredoxin 2 (Fig 5). Taken together, these data suggest that the reduction of ximelagatran by mARC2 in the outer mitochondrial membrane lead to the formation of a product(s) that disturb the redox balance in mitochondria. Indeed, a number of hepatotoxic drugs associated with specific HLA alleles have been found to be mitotoxic [19]. It can be suggested that the mARC2 redox system in the outer mitochondrial membrane may metabolically activate different hepatotoxic drugs, for which the mechanistic basis of their mitotoxicity remains unknown (cf. [19]). Further studies are warranted that should analyze in more detail the basis for the mARC2 and ximelagatran induced redox changes as well as the link to HLA specificity among the patients suffering from such toxicity.

In summary, we have provided evidence for nutritional regulation of the mARC2 enzyme in the animal and human liver and the distribution of this enzyme in human liver and fat, critical tissues for control of human lipogenesis as well as the involvement of mARC2 in the reduction of ximelagatran to the putative metabolite(s) affecting the redox properties of mitochondria. It might be suggested that mARC2 could be chosen as a future drug target for the control of lipid homeostasis.

## Supporting Information

S1 Fig mARC1 and mARC2 gene expression in human fetal and adult liver. mARC1 and mARC2 gene expression in human fetal (n = 14) and adult (n = 88) liver samples was determined by microarray analysis and presented as background corrected probe intensities.
(TIF)

S2 Fig Fatty acid composition of triglycerides (TG) in adipocytes from control and siRNA mARC2 knock down cells. Levels of lipid species reflect integrated areas of mass chromatograms as determined by high resolution Orbitrap LC-MS/MS from control and siRNA mARC2 knock down adipocytes (n = 6 each) in a non-targeted approach.
(TIF)

S3 Fig Fatty acid composition of phospholipids in adipocytes from control and siRNA mARC2 knock down cells. Phospholipids are represented by phosphatidylcholine (PC), phosphatidylethanolamine (PE), alkyl-phosphatidylethanolamine (aPE), phosphatidylglycerol (PG), phosphatidylinositol (PI) and phosphatidylserine (PS) species. Data is represented as indicated in S2 Fig.
(TIF)
