# Supplementary figures and images for: Expression and Function of mARC: Roles in Lipogenesis and Metabolic Activation of Ximelagatran
Source: PLoS One. 2015 Sep 17;10(9):e0138487. doi: 10.1371/journal.pone.0138487 (PMC4574727; doi:10.1371/journal.pone.0138487)

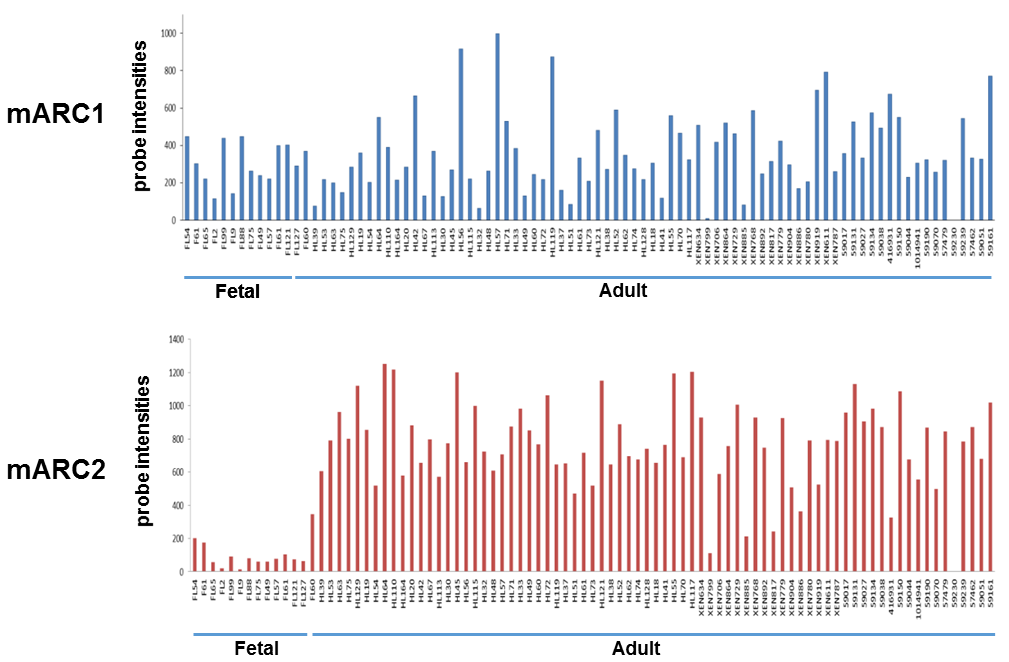

Supplement: S1 Fig — mARC1 and mARC2 gene expression in human fetal (n = 14) and adult (n = 88) liver samples was determined by microarray analysis and presented as background corrected probe intensities. (TIF) [file pone.0138487.s001.tif]

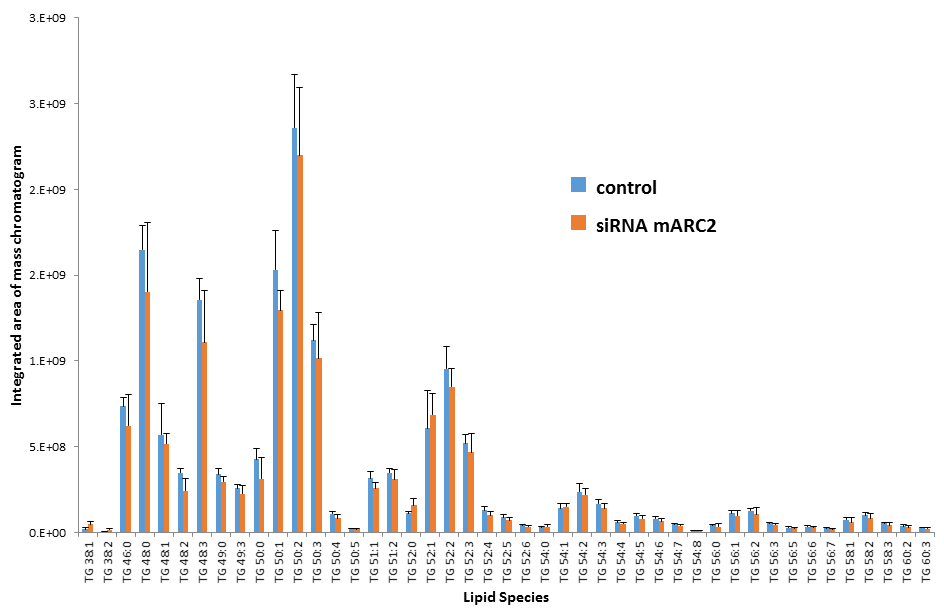

Supplement: S2 Fig — Levels of lipid species reflect integrated areas of mass chromatograms as determined by high resolution Orbitrap LC-MS/MS from control and siRNA mARC2 knock down adipocytes (n = 6 each) in a non-targeted approach. (TIF) [file pone.0138487.s002.tif]

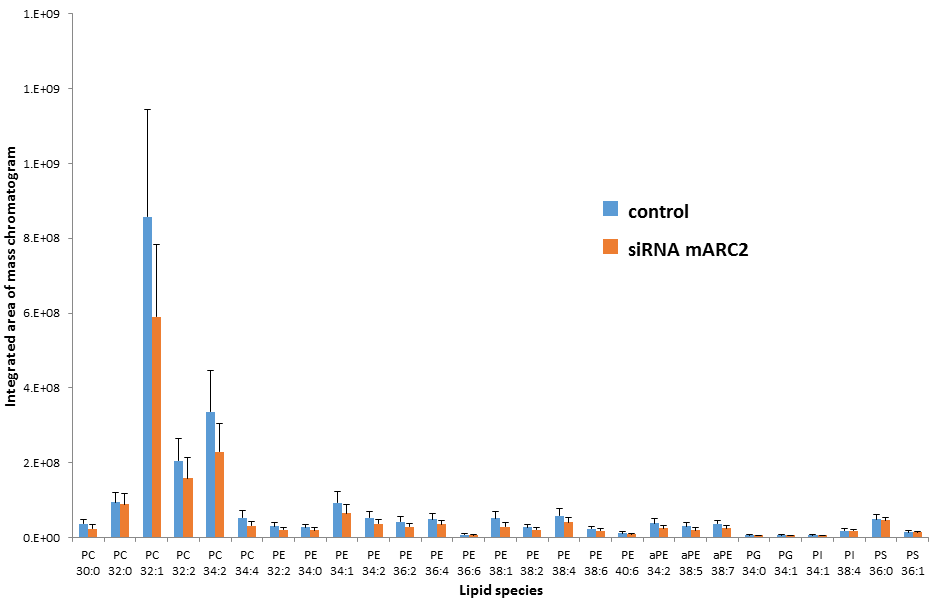

Supplement: S3 Fig — Phospholipids are represented by phosphatidylcholine (PC), phosphatidylethanolamine (PE), alkyl-phosphatidylethanolamine (aPE), phosphatidylglycerol (PG), phosphatidylinositol (PI) and phosphatidylserine (PS) species. Data is represented as indicated in S2 Fig. (TIF) [file pone.0138487.s003.tif]
